# Social traits modulate attention to affiliative cues

**DOI:** 10.3389/fpsyg.2014.00649

**Published:** 2014-06-24

**Authors:** Sarah R. Moore, Yu Fu, Richard A. Depue

**Affiliations:** Department of Human Development, Cornell UniversityIthaca, NY, USA

**Keywords:** attention, emotional processing, social closeness, affiliation, attachment

## Abstract

Neurobehavioral models of personality suggest that the salience assigned to particular classes of stimuli vary as a function of traits that reflect both the activity of neurobiological encoding and relevant social experience. In turn, this joint influence modulates the extent that salience influences attentional processes, and hence learning about and responding to those stimuli. Applying this model to the domain of social valuation, we assessed the differential effects on attentional guidance by affiliative cues of (i) a higher-order temperament trait (Social Closeness), and (ii) attachment style in a sample of 57 women. Attention to affiliative pictures paired with either incentive or neutral pictures was assessed using camera eye-tracking. Trait social closeness and attachment avoidance interacted to modulate fixation frequency on affiliative but not on incentive pictures, suggesting that both traits influence the salience assigned to affiliative cues specifically.

## Introduction

Visual attention is imperative to the selection of inputs of value to be processed in depth, contributing to planning and controlling one's interaction with the environment (Knudsen, 2007; Chun et al., 2011; Kaspar and König, 2012). It has become clear that emotional-motivational cues are prioritized by neural systems that permit access to attention due to their adaptive value (Dolan, 2002; Vuilleumier, 2005). For example, stimuli previously associated with reward can drive attentional capture even when a stimulus is no longer relevant to an ongoing task and lacks physical salience (Anderson et al., 2011, 2013; Anderson and Yantis, 2012). Indeed, a robust literature reports that emotional cues are more likely than neutral cues to guide attention in visual search and spatial orienting tasks (Mogg et al., 1997; Ohman et al., 2001; Armony and Dolan, 2002). Thus, neural processes rendering heightened signal strength to valenced cues drive the sensitivity of the attentional system to emotional events (Vuilleumier, 2005).

Studies of patients with hemispatial neglect following stroke-induced damage to the parietal cortex indicate that attentional capture of emotional cues occurs when these cues are presented to the normally neglected (i.e., unattended) visual region, despite the inability of non-emotional cues to be similarly detected if presented in the same region (Vuilleumier and Schwartz, 2001; Tamietto et al., 2007). This work indicate a role of subcortical emotional mechanisms outside of awareness in the detection of affective cues, and the subsequent sensitization of visual attentional networks to this information.

Subcortical emotional networks that establish the affective significance of visual inputs include the amygdala, a structure essential to the unconscious evaluation of emotionally salient information (Balleine and Killcross, 2006). Affective encoding of crude visual representations in the basolateral complex of the amygdala (BLA) can occur within milliseconds via a subcortical visual pathway from the superior colliculus through the medial pulvinar to the BLA, outside of sensory awareness (Tamietto and de Gelder, 2010). If the input is determined by the BLA to be of affective significance, then BLA excitatory backprojections to all stages of sensory processing pathways activate visual (and other sensory) networks to provide a biased processing of the emotional input while inhibiting the representation of other, less salient stimuli (Kapp et al., 1992; Aston-Jones et al., 1999; Amaral et al., 2003; Zald, 2003). Amygdala activations to faces rendered salient through expressional transfiguration, but not to normal faces, are continuous across repeated presentations, thus creating a sustained biasing effect (Rotshtein et al., 2001). Moreover, BLA backprojections to many cortical and subcortical regions can bias processing in attentional networks, and enhance affective memory consolidation and retrieval in the hippocampus, all aimed toward deeper processing of affective cues in the environment. Overall, then, affective encoding by the BLA informs downstream attention processes in a prolonged manner in order to promote orientation and sustained attention to salient affective information (Morris et al., 1998a,b; Holland and Gallagher, 1999; Anderson and Phelps, 2001; Vuilleumier, 2002; Phelps and LeDoux, 2005; see Kaspar and König, 2012 for a more detailed review of these attentional neural networks).

Although emotionally-laden stimuli are preferred in general for access to attentional processing, the current emotional state of an individual may also bias processing of and attention to affective cues. A large literature indicates that emotional-motivational states can determine the extent to which particular cues are deemed salient and, thereby, are selected for attentional processing to initiate adaptive responses. For example, negative mood states increase attention to aversive stimuli to ensure avoidance of potential danger (Gilboa-Schechtman et al., 2000; Mathews et al., 2003; Bar-Haim et al., 2007). Thus, the guidance of attention is driven by both the affective nature of environmental stimuli as well as by the salience attributed to those stimuli based on the emotional state of the observer.

A similar but more enduring influence of emotion on attention derives from personality traits. Prominent models suggest that personality traits reflect the activity of emotional-motivational systems that evolved to increase adaptation to broad classes of stimuli associated with positive and negative outcomes, such as incentive and aversive stimuli. Individual differences in these systems are then theorized to reflect variation in the sensitivity to corresponding affective stimuli (Gray, 1973; Cloninger, 1986; Depue and Collins, 1999; Gray and McNaughton, 2000; Depue and Morrone-Strupinsky, 2005; Depue and Fu, 2013).

Most relevant to our discussion is that individual differences in sensitivity to critical affective stimuli would, over time, yield enduring biases in the affective encoding of corresponding stimuli by the BLA and, hence, more frequent activation of the corresponding affective state. For instance, an individual high in trait anxiety may encode stimuli as salient that have an even weak relation to threat (i.e., manifest a reduced threshold for assigning affective significance to potential threats) (Canli, 2008; Depue and Fu, 2012). Indeed, Canli (2004) demonstrated that amygdala activation in response to fearful and happy faces corresponded to trait levels of neuroticism and extraversion, respectively. This enhancement of affective encoding of environmental stimuli may be one contribution to an enduring down-stream biasing of perception, attention, and memory toward negative stimuli in studies of neuroticism and toward positive incentive stimuli in studies of extraversion (Derryberry and Reed, 1994; Canli, 2004, 2008; Knutson and Bhanji, 2006; Bar-Haim et al., 2007; Ponari et al., 2013). Such enhanced affective encoding at subcortical levels of sensory processing could have a substantial, enduring influence on attention by top-down cognitive processes involving central representations of affective (negative or positive) outcome expectations held in working memory (Depue and Morrone-Strupinsky, 2005; Canli, 2008).

Very little empirical work has been devoted to the influence of socially-relevant traits, namely personality and attachment style, on attentional processes to affiliative cues. In terms of personality, two traits seem more relevant to social valuation and are worth distinguishing in this respect. The trait of extraversion concerns sensitivity to incentive reward stimuli in general, only a portion of which are social in nature (Digman, 1990; Depue and Collins, 1999; Depue and Morrone-Strupinsky, 2005; Smillie, 2013). In the social realm, extraversion as incentive motivation describes the tendency to be socially dominant, and more generally is related to a sense of potency in accomplishing goals (whether social or unsocial in nature) and to an activated positive affective experience of elation, enthusiasm, optimism, and euphoria (Depue and Collins, 1999; Tellegen and Waller, 2008; Depue and Fu, 2013). In contrast, the trait of social closeness (SC), which partially overlaps traits of communion, affiliation, and agreeableness (Depue and Morrone-Strupinsky, 2005; Tellegen and Waller, 2008), defines the extent to which an individual values and enjoys close social contact vs. being alone, and feels and displays warmth, affection, and calm gratification in social interactions. Both trait agentic extraversion and affiliation predict increased feelings of vigor and excitement in response to an appetitive mood induction involving multi-modal pleasant vignettes of both social and non-social scenarios (Smillie et al., 2013). In contrast, the distinctness of the affective nature of extraversion and SC was demonstrated in a study of film-induced affective experience using two clips of specific positive experiences. On the one hand, SC but not extraversion was significantly correlated with the warmth and affection generated by a film showing affectionate interpersonal ties; whereas, on the other, extraversion but not SC was significantly related to increased positive activation, elation, and enthusiasm in response to a film illustrating triumphant success in striving to win a football game (Morrone-Strupinsky and Depue, 2004).

Although adequate trait levels of SC are believed to be essential to the formation and maintenance of social bonds (Depue and Morrone-Strupinsky, 2005; Tellegen and Waller, 2008), its influence on attention to social cues has not been studied. Perhaps SC modulates attentional processes through an influence on affective encoding as theorized for neuroticism and extraversion above. In any case, the influence of SC on attention to affiliative stimuli is important to determine, because attentional modulation by trait SC would likely influence the extent to which individuals focus on and gravitate toward their interpersonal environment in establishing social bonds.

Finally, the extent of positive valuation of affiliative stimuli might also be influenced by early experience with primary caregivers and close friends–the quality of which is thought to influence the development of attachment styles (Bowlby, 1982). Two orthogonal dimensions, anxiety and avoidance are theorized to reflect internal working models of the self and others (Brennan et al., 1998). Anxiety involves a working model of *the self* as unworthy of the care and responsiveness of others, leading to a hyperactive strategy for heightened vigilance for rejection, abandonment, and unavailability of attachment figures. Avoidance reflects working models of *others* as incapable of providing comfort and as unresponsive to one's needs. In contrast to anxiety, an avoidant attachment strategy involves deactivation or downregulation of systems detecting and encoding attachment information to ensure no dependence on or vulnerability to attachment figures.

Research linking anxiety and avoidance to attention to attachment-related cues reported (i) altered emotional processing of and attention to attachment cues (Mikulincer et al., 2000, 2002; Gillath et al., 2005), and (ii) that attachment avoidance imparts disengagement from cues depicting sadness and distress that would normally elicit comfort and proximity-seeking behavior (Kirsh and Cassidy, 1997; Dewitte et al., 2007; Suslow et al., 2010). Similar to the theorized influence of personality traits on attentional processes, it has been suggested that patterns of neural processing and attention, even to attachment cues presented outside of attentional awareness (Suslow et al., 2009), are due to subcortical mechanisms that deploy or divert attentional processing toward or away from relevant attachment information (Niedenthal et al., 2002).

Given that attachment avoidance involves both negative appraisals of social others and disengagement from intimacy and closeness, it follows that this strategy could be particularly relevant to the subconscious valuations of social stimuli that guide attention. Perhaps then, the guidance of attention toward (or away from) affiliative cues involves both (i) the trait level of affective encoding of affiliative cues corresponding to SC, and (ii) the extent that persistent low positive affective encoding of social stimuli consistent with avoidant attachment style has developed through interpersonal experiences of rejection, abandonment or neglect. Thus, we theorize that the effect of SC on the guidance of attention to affiliative cues (and proximately social behavior) will be mediated by the degree of avoidant attachment due to the role of early and ongoing attachment experiences in adjusting the valuation and affective encoding of affiliative others.

The current study applied camera eye-tracking to first assess the relations of SC and attachment to attentional focus by affiliative stimuli as a means of gauging the role of these traits in modulating attention through affective encoding. To ensure assessment of attentional bias to pictures depicting affiliative value specifically, (rather than a bias to any emotionally-valenced stimulus or to positive stimuli), we displayed pictures of individuals in affiliative interactions paired side-by-side with pictures of individuals in either neutral contexts or in activities of high incentive but low affiliative value. We hypothesized that the duration of time spent attending to affiliative pictures in the presence of neutral and incentive distractors would relate positively to trait levels of SC, but negatively to avoidant attachment patterns.

Second, to shed light on the paths of socio-emotional influence in attentional behavior, we tested the hypothesis that the effect of trait levels of SC on attention to affiliative cues is mediated by attachment avoidance. Specifically, we expected that the effect of SC on attention to affiliative cues in particular could be accounted for by level of avoidant attachment (i.e., a significant indirect effect). If our theoretical model is correct, this finding would lend support to the notion that affiliative cues are assigned heightened salience and drive attentional processes to a greater degree in individuals who (i) highly value SC and experience pleasure from interpersonal contact, and (ii) do not subsequently perceive such cues from an avoidant self-schema due to attachment experiences.

## Materials and methods

### Participants

The intended sample size of at least 50 participants was chosen based on a priori power analysis indicating that this sample would yield 85% power to detect an effect of SC or avoidance on attention to affiliative cues (see Analysis section). Participants were Cornell University students recruited by email sent to freshman undergraduate females for participation in a multi-study project. A purely female sample was selected due to the nature of the affiliative stimuli (which include depictions of close romantic, and parent-child embraces) to reduce noise, as any potential gender difference was not of interest to the study's research goals. From those that responded (*N* = 783), 70 were selected randomly. Of the 70 selected, 62 agreed to participate in the current study, and 5 of these were excluded from analysis due to equipment failure. This resulted in a final group of 57 females [*M*(age) = 20.48 y]. The study was approved by Cornell University's Institutional Review Board for Human Participants. Participants provided written informed consent upon arrival at the lab.

### Visual stimuli

Three types of affective pictures were selected as stimuli: neutral, affiliative, and incentive. The 24 neutral, non-emotional pictures were of men and women with non-emotional facial expressions from the International Affective Picture System (IAPS; Lang et al., 2008), which provides pictures with standardized ratings of emotional valence and arousal. The neutral pictures had a mean emotional valence rating of 1.1, and a mean arousal rating of 1.2.

The affiliative and incentive pictures were selected to elicit feelings of warmth, affection, and caring vs. elation, enthusiasm, and excitement, respectively. Because the original selection of the IAPS pictures did not focus on the constructs of affiliative and incentive affect when constructed, we selected pictures that represented both of these affect types mainly from web sites, and a few from the IAPS. Having constructed and validated video material assessing these two affective constructs (Morrone-Strupinsky and Depue, 2004), and having considered their phenomenology previously (Depue and Collins, 1999; Depue and Morrone-Strupinsky, 2005), we used these specific constructs as a guide in selecting the pictures, although we purposely selected broadly from the constructs. We arrived at 50 affiliative and 50 incentive affect pictures. Then, 150 participants rated each of these 100 pictures for three affects, each designated by three adjectives: (1) affiliative (*warm, affectionate, caring*), (2) incentive (*excited, enthusiastic, elated*), and (3) negative affect (*nervous, tense, worried*), counterbalanced for order of rating across pictures. These adjectives were selected from those used in the PANAS scales (for positive incentive affect and negative affect), and from those that mark the high end of a Communion dimension (Morrone-Strupinsky and Depue, 2004; Depue and Morrone-Strupinsky, 2005). Ratings of each of the three sets of adjectives were completed on a 5-point rating scale: *none, a little, moderately, strong*, and *very strong*. Participants were instructed to look at each picture (shown via powerpoint for 6 s), and then to rate how much the picture made them “feel” each of the three affects, using the 5-point rating scale for each.

From this assessment, 24 affiliative pictures were selected that had (i) the highest affiliation ratings (mean = 4.0 or higher), plus (ii) a 2.5 or lower mean rating for incentive affect, and (iii) a 1.0 or lower mean rating for negative affect. The affiliative pictures consisted of mothers and/or fathers with infants or children, children hugging or smiling broadly, and couples involved in non-sexual, caring touch. In addition, 24 incentive pictures were selected that had (i) the highest incentive ratings (mean = 4.0 or higher), plus (ii) a 2.5 or lower mean rating for affiliative affect, and (iii) a 1.0 or lower rating for negative affect. The incentive pictures consisted of people involved in exciting scenes from various sports activities.

**Figure 1:**
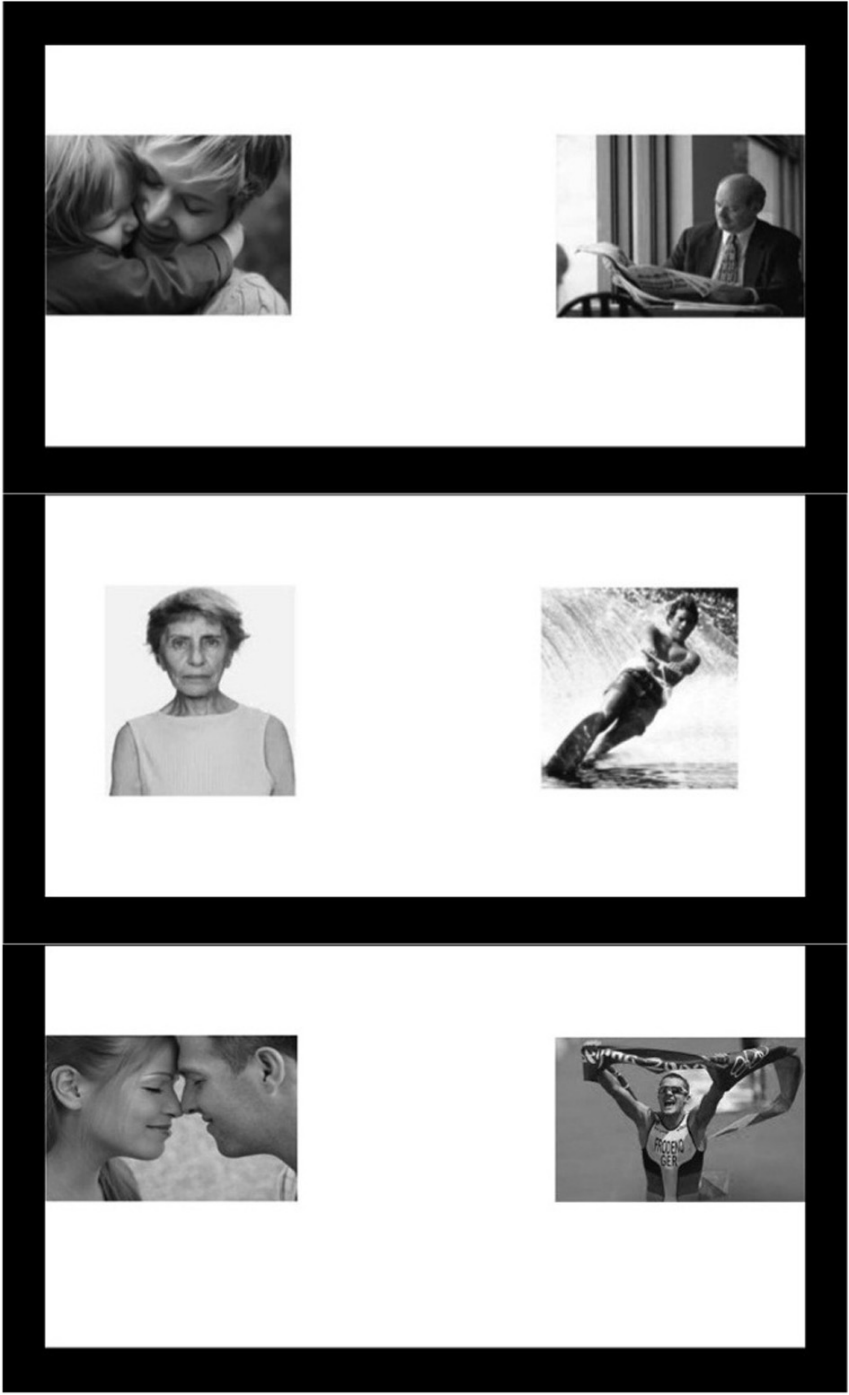
**Example of pictures used in each of three combination-conditions**. (**Top**: affiliative vs. neutral; **Middle**: incentive vs. neutral; **Bottom**: affiliative vs. incentive).

The 72 final pictures were presented in pairs side-by-side in three combination-conditions: 12 affiliative vs. 12 neutral; 12 incentive vs. 12 neutral; and 12 affiliative vs. 12 incentive (see Figure 1 for examples). Pairs of pictures were matched based on perceptual complexity, luminance, and size (standard 3.5 × 4 inches) using the GNU Image Manipulation Program, and spaced equidistant from the center of the image. Within each combination, the affect pictures were presented half on the right and half on the left (e.g., 6 affiliative on the right vs. neutral, and 6 affiliative on the left vs. neutral). None of the pictures was repeated, and all picture pairs were presented in randomized order.

### Eye-tracking

Humans scan a visual scene by directing their gaze to a number of points of interest, and briefly “fixating” on those points. Fixation points of a visual scene are obtained through the fovea, which provides the highest-resolution input to the visual system. Visual fixations entail the active acquisition of visual information, information processing, and visuospatial attention (Yarbus, 1967; Hoffman and Subramaniam, 1995; Kowler et al., 1995; Deubel and Schneider, 1996; Peterson et al., 2004). Between fixations on a visual scene, rapid saccadic eye movements serve to locate attentional targets. During the 50–80 ms in which saccades occur, visual information processing is suppressed, and visual perception is reduced (Ross et al., 2001). Hence, we assessed the most common parameters used in eye-movement studies: (i) number of fixations, (ii) mean fixation duration, and (iii) mean dwelling time (i.e., total time spent fixating on a target; Jacob and Karn, 2003).

The Eyelink 1000 (SR Research) high-speed camera eye-tracking system was used to record eye movements through pupil-center corneal reflection. Eyelink 1000 provides high spatial resolution and a 500-Hz sampling rate (2-ms sampling resolution). Participants sat in a dimly lit (500 Lux), soundproof chamber facing a 29” monochrome computer monitor (38” × 29”, or 18 × 13.5 cm) with a screen resolution of 1024 by 768 pixels, with the gaze tracker located below the screen. Participants sat in a chair, and their head was comfortably positioned in a head-mount positioned on a table located 500 mm from the camera lens. The head-mount was used to position and hold stable the face to ensure highly accurate monocular data acquisition (average accuracy 0.15°). A participant's gaze [using the left eye, with eyeglasses (but not contact lenses) if typically worn] was calibrated to the eye-tracker by collecting fixation samples from known target points in order to map raw eye data to gaze position, and then gaze accuracy was validated through displaying a second set of known targets. The head's exact position was automatically recorded by measuring the distance from the lens to the forehead. Visual stimuli were presented using the Eprime software package (Psychological Software Tools, Inc.).

A trial consisted of (i) the presentation of a central fixation point (a black “+,” 0.63” × 0.63”) on the monitor for 2 s, with a beep (60 dB) sounding at the second-half of the fixation to alert the participant to the imminent presentation of the visual stimuli, (ii) display of paired pictures (the pair = 5” diameter) for 8 s, and (iii) a randomized interval of 1.5, 2, or 2.5 s (during which the screen was illuminated at the same intensity as the screen's background illumination during picture displays).

Raw eye-position data were analyzed in Python to assess dependent eye-movement variables, including fixation frequency and the total fixation time on the left- and right-positioned pictures. Consistent with previous research (Caseras et al., 2007; Rauthmann et al., 2012), a fixation was defined as a stable gaze at a point of interest for at least 100 ms. No significant differences on any of the variables were found between left and right positioning of affect pictures. Therefore, to best summarize the total of 12 displays of paired pictures for each of the three combinations (affiliative vs. neutral, incentive vs. neutral, and affiliative vs. incentive), data were combined across left and right positions. For each of the paired pictures, the average fixation frequency, the total fixation time, and the percent of fixation time on each picture was calculated. Because the average fixation frequency and total fixation time were so highly correlated (*r* = 0.91), only the average fixation frequency is discussed (indeed, analysis of both variables showed practically identical results).

### Measures

#### Social closeness scale

The Social Closeness scale is one of the four major higher-order trait scales in Tellegen's Multidimensional Personality Questionnaire (MPQ; Tellegen and Waller, 2008), consisting of 22 true-false items. It assesses the extent to which an individual values close interpersonal relations and social contact. High scores indicate that the individual is sociable, likes to be with people, takes pleasure in and values close personal ties, is warm and affectionate, and turns to others for comfort and help. The scale has an alpha = 0.94, stability over 3 months = 0.88, and is correlated 0.89 with the Agreeableness scale of the NEO-PI (Tellegen and Waller, 2008).

#### Experience in close relationships scale (ECR)

The ECR assesses attachment security in adult romantic relationships, where secure attachment is reflected by lower scores on two orthogonal dimensions, anxiety and avoidance (Brennan et al., 1998). The anxiety dimension includes 18 items assessing fear of interpersonal rejection or abandonment, an excessive need for approval from others, and feelings of distress caused by the unavailability or unresponsiveness of a partner. The avoidance dimension also has 18 items and assesses fear of dependency and interpersonal intimacy, an excessive need of self-reliance, and reluctance to self-disclose. The ECR demonstrates high internal consistency and test–retest reliability over several months, as well as sound discriminant and convergent validity with other scales (Brennan et al., 1998). Participants are instructed to rate the items from 1 (strongly disagree) to 7 (strongly agree) based on their experiences in close relationships in general.

### Procedure

After being positioned in the chamber, participants were instructed via audio recording about the nature of the task, and were told to “view the pictures naturally.” Six practice trials were first presented, which consisted of the presentation of pairs of neutral stimuli not used in the test trials. All participants performed accurately during the practice trials. Participants were then given the opportunity to ask any final questions before beginning the test trials. Then, 36 test trials of paired pictures were presented. There was a 1-min rest period after the first 18 trials. Participants filled out the SC scale during recruitment, and the ECR after the eye-tracking task.

### Analysis

To gauge whether individual difference measures related to attention to affiliative cues, we conducted repeated measures analysis of covariance (ANCOVA). Dimensional trait measures (SC or attachment dimensions), and picture-type (categorical variable; repeated measure) served as independent variables that predicted the attention variable of fixation frequency for each of the three picture combination-conditions (affiliative vs. neutral, incentive vs. neutral, and affiliative vs. incentive). A repeated-measures model is necessary to account for the correlation between fixation frequencies on the two picture types within each subject. Multi-level models also account for such dependencies in nested data sets, and multi-level analyses yielded the same estimates and standard errors [nlme package (Pinheiro et al., 2014) in R (R Development Core Team, 2010)]. We report the repeated measure model results due to the ease of interpreting and reporting the main results and the follow-up interaction effects for the reader.

In these models, we tested for the presence of an interaction effect between SC or attachment (modeled separately due to a high correlation between SC and avoidance; *r* = −0.69, *p* < 0.01) and the picture-type (e.g., affiliative vs. neutral). We predicted that the effect of picture-type on fixation frequency would vary according to SC (or attachment scores) in the affiliative-neutral and affiliative-incentive conditions, but not in the incentive-neutral condition. This pattern, where the relation of social valuation measures to fixation frequency depends on the presence of affiliative but not incentive pictures, would support the proposition that SC (or avoidant attachment) modulation of attention is specific to affiliative cues. If both SC and avoidance demonstrated significant (but opposing) effects on attention to affiliative stimuli, a final test of moderated mediation would be performed (see below).

It is important to note that the detection of moderation effects can often be difficult in the context of field studies, in which very large samples are often required for sufficient power to detect effects (McClelland and Judd, 1993). In the current study, control over the pictures presented to participants enables the detection of an interaction between two stochastically independent variables (SC or attachment and picture-type). That is, we are not restricting the range of effects by estimating a product term, as participants with varying levels of individual difference scores viewed fixed picture pairs. By controlling the characteristics of the pictures (i.e., perceptual complexity, luminance, and size) and manipulating the affective content, any effect of personality on fixation frequency is expected to emerge in the interaction term, which captures whether the relation between personality and fixation frequency differs between picture type. Based on this design, we ran a Monte Carlo power analysis for the interaction effect. Simulations consisted of randomly sampling a variable from the normal distribution (i.e., personality), modeled to have an opposing effect on the number of fixations for picture type 1 vs. picture type 2. The magnitude of this effect is inconsequential, as it is the opposite sign (e.g., −0.1 effect on neutral vs. 0.1 effect on affiliative; R code and results available on request), which drives the interaction effect. The repeated measures ANCOVA was fit to return the *p*-value of the interaction term. Finally, the simulation was ran 5000 times for sample sizes ranging from 50 to 100 in increments of 5, and the *p*-value was averaged and subtracted from 1 to obtain the power estimate for each sample size. Since a sample of 50 yielded 85% power (which gradually increased to 96% with a sample of 100), we aimed to achieve a sample of 50 or above.

## Results

Descriptive statistics for eye-movement parameters and trait scales are provided in Table 1, where the attention parameter for each picture–the average number of fixations–is summarized for the three conditions (affiliative vs. neutral, incentive vs. neutral, and affiliative vs. incentive). In Table 2, intercorrelations between trait scores and number of fixations for each picture-type (affiliative, neutral, incentive) are shown. Centered results for the final six models testing SC and attachment separately for the three conditions are summarized in Table 3.

**Table 1:** **Descriptive statistics for eye movement parameters and trait scales**.

**Variables**	***M***	***SD***
**AFFILIATIVE vs. NEUTRAL CONDITION**		
Fixation frequency affiliative	10.17	2.13
Fixation frequency neutral	6.14	1.69
Percent fixations on affiliative	0.62	0.08
Total fixation duration (ms)	4396.97	793.45
**INCENTIVE vs. NEUTRAL CONDITION**		
Fixation frequency incentive	9.22	1.87
Fixation frequency neutral	7.48	1.45
Percent fixations on incentive	0.55	0.08
Total fixation duration (ms)	4380.76	828.76
**AFFILIATIVE vs. INCENTIVE CONDITION**		
Fixation frequency affiliative	9.33	1.79
Fixation frequency incentive	7.18	1.64
Percent fixations on affiliative	0.56	0.08
Total fixation duration (ms)	4415.70	858.53
**TRAIT MEASURES**		
Social closeness	15.74	6.44
Attachment anxiety	3.62	1.13
Attachment avoidance	3.02	1.16
*N* = 57		

**Table 2:** **Correlations among variables**.

**Variables**	**Affiliative vs. neutral condition**			**Incentive vs. neutral condition**			**Affiliative vs. incentive condition**			**Trait measures**		
	**1**	**2**	**3**	**4**	**5**	**6**	**7**	**8**	**9**	**10**	**11**	**12**
**Eye movement parameters**												
**AFFILIATIVE vs. NEUTRAL CONDITION**												
1. Fixation frequency Affiliative	–											
2. Fixation frequency neutral	−0.06	–										
3. Percent fixations on affiliative	0.67^**^	−0.77^**^	–									
**INCENTIVE vs. NEUTRAL CONDITION**												
4. Fixation frequency incentive	0.69^**^	0.27^*^	0.27^*^	–								
5. Fixation frequency neutral	0.24	0.47^**^	−0.13	−0.12	–							
6. Percent fixations on incentive	0.33^**^	−0.08	0.23	0.78^**^	−0.70^**^	–						
**AFFILIATIVE vs. INCENTIVE CONDITION**												
7. Fixation frequency affiliative	0.78^**^	0.12	0.45^**^	0.49^**^	0.41^**^	0.11	–					
8. Fixation frequency incentive	0.27^*^	0.67^**^	−0.29^*^	0.49^**^	0.32^*^	0.17	0.04	–				
9. Percent fixations on affiliative	0.35^**^	−0.38^**^	0.51^**^	0.00	0.10	−0.08	0.69^**^	−0.68^**^	–			
**TRAIT MEASURES**												
10. Social closeness	0.20	−0.50^**^	0.50^**^	0.00	−0.19	0.11	0.08	−0.28^*^	0.26^*^	–		
11. Attachment anxiety	−0.08	0.05	−0.08	−0.15	0.19	−0.23	−0.01	0.07	−0.04	−0.12	–	
12. Attachment avoidance	−0.24	0.37^**^	−0.42^**^	−0.01	0.01	−0.01	−0.15	0.24	−0.28^*^	−0.69^**^	0.28^*^	–

* p < 0.05;

** *p < 0.01*.

**Table 3:** **Repeated measures ANCOVA results**.

	**Affiliative vs. neutral condition**		**Incentive vs. neutral condition**		**Affiliative vs. incentive condition**	
	***B***	***SE (B)***	***B***	***SE (B)***	***B***	***SE (B)***
**SOCIAL CLOSENESS MODELS**						
Intercept	2.01^**^	0.24	0.87^**^	0.22	1.08^**^	0.22
Social Closeness	0.09^**^	0.04	0.01	0.03	0.04	0.04
Picture	−4.03^*^	0.34	−1.73^**^	0.32	−2.16^**^	0.32
Social Closeness × Picture	−0.22^**^	0.05	−0.05	0.05	−0.11^*^	0.05
Adjusted *R*^2^	0.58^**^		0.20^**^		0.30^**^	
**ATTACHMENT MODELS**						
Intercept	2.01^**^	0.24	0.87^**^	0.22	1.08^**^	0.23
Anxiety	−0.12	0.23	−0.35	0.21	−0.02	0.21
Avoidance	−0.58^**^	0.22	−0.02	0.20	−0.36	0.20
Picture	−4.03^**^	0.34	−1.73^**^	0.31	−2.16^**^	0.32
Anxiety × Picture	−0.03	0.32	0.58^*^	0.29	−0.02	0.30
Avoidance × Picture	1.12^**^	0.31	−0.10	0.28	0.68^**^	0.29
Adjusted *R*^2^	0.56^**^		0.21^**^		0.29^**^	

* p < 0.05;

** *p < 0.01. Displaying results of fully centered regression; Picture-type coded with first picture listed in condition as 0 (reference group) and second picture as 1*.

### SC and attention

Repeated measures ANCOVA assessed whether SC and picture-type (e.g., affiliative or neutral) significantly predicted fixation frequency for each of the three picture conditions. These models test whether the effect of picture-type on attention depends on SC (SC × picture-type interaction).

**Figure 2:**
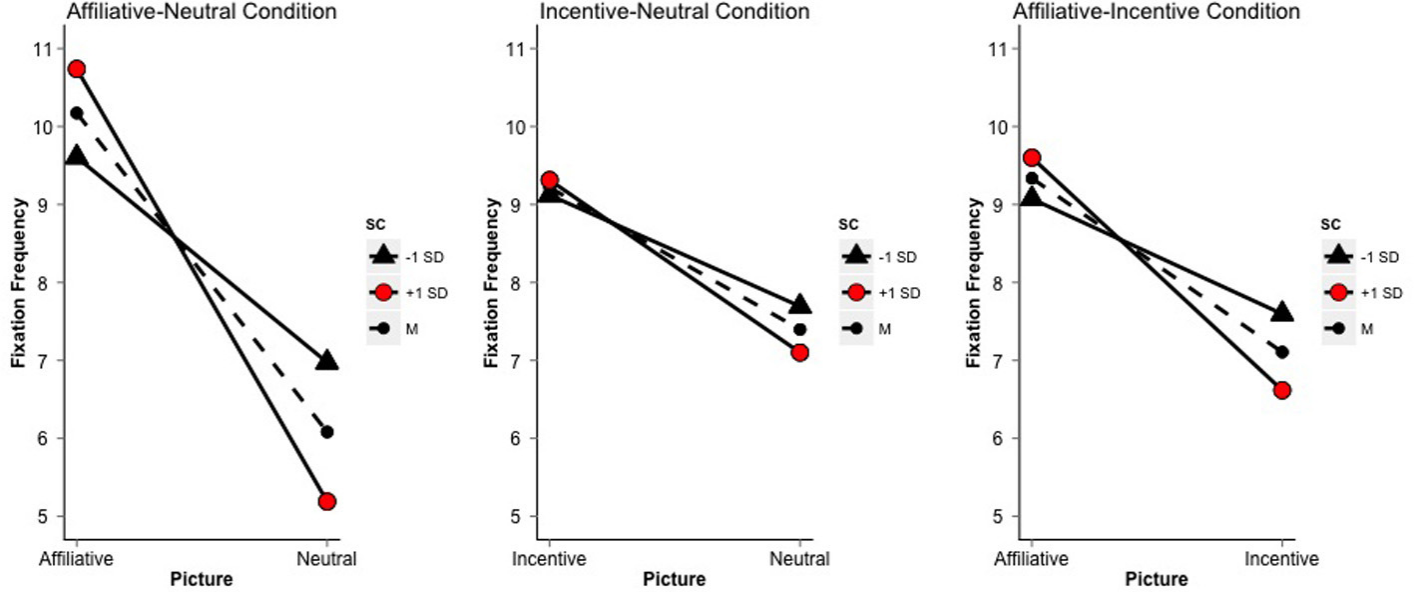
**Simple slopes of picture on fixation frequency for ±1 *SD* and the mean (dashed line) of SC**.

In the affiliative-neutral condition, the repeated measures ANCOVA of fixation frequency by picture-type and SC indicated that picture-type, SC, and the interaction of picture-type and SC accounted for a significant amount of the variance in number of fixations [*R*^2^_adjusted_ = 0.58, *F*_(3, 110)_ = 53.66, *p* < 0.01]. There was a significant interaction of picture-type by SC. Specifically, as shown in Figure 2, simple slopes of SC for each picture type indicated that SC positively relates to fixation frequency on the affiliative picture [*b* = 0.09, *t*_(110)_ = 2.34, *p* < 0.05], but negatively relates to fixation frequency on the neutral picture [*b* = −0.13, *t*_(110)_ = −3.53, *p* < 0.01]. The simple slopes of picture-type on fixation frequency demonstrated that, on average, for low SC scorers (−1 *SD*), there were 2.61 more fixations on the affiliative picture compared to the neutral picture [*t*_(110)_ = −5.41, *p* < 0.01], while for high SC scorers (+1 *SD*), there were 5.43 more fixations on the affiliative picture than on the neutral picture [*t*_(110)_ = −11.39, *p* < 0.01] (Figure 2), indicating that as SC score increased, attention was increasingly drawn to the affiliative picture opposed to the neutral picture.

In the incentive-neutral regression, picture-type, SC, and the interaction of picture-type and SC accounted for a significant amount of the variance in number of fixations [*R*^2^_adjusted_ = 0.20, *F*_(3, 110)_ = 10.45, *p* < 0.01]. The interaction of SC with picture-type was not statistically significant (Figure 2), indicating that the number of fixations on incentive vs. neutral pictures did not depend on SC.

In the affiliative-incentive regression, picture-type, SC, and their interaction accounted for a significant amount of the variance in number of fixations [*R*^2^_adjusted_ = 0.30, *F*_(3, 110)_ = 17.01, *p* < 0.01]. As shown in Figure 2, the interaction of picture-type by SC was significant, such that the simple slope of SC on fixation frequency was positive for the affiliative picture [*b* = 0.04, *t*_(110)_ = 1.17, *p* < 0.05], but negative for the incentive picture [*b* = −0.07, *t*_(110)_ = −1.91, *p* = 0.06]. The simple slopes of picture-type on fixation frequency showed that for low SC scorers (−1 *SD*), there were 1.46 more fixations on the affiliative picture than the incentive picture [*t*_(110)_ = −3.24, *p* < 0.01], while for high SC scorers (+1 *SD*), there were on average 2.85 more fixations on the affiliative picture than on the incentive picture [*t*_(110)_ = −6.33, *p* < 0.01] (Figure 2). These results indicate that, as SC scores increased, attention was increasingly drawn to the affiliative picture from the incentive picture.

### Attachment dimensions and attention

Repeated measures ANCOVA assessed whether attachment avoidance, attachment anxiety, and picture-type significantly predicted fixation frequency for each of the three picture conditions. Similar to the SC models, these analyses assess whether the effect of picture-type on attention depends on attachment dimensions (avoidance × picture-type, and anxiety × picture-type interactions).

**Figure 3:**
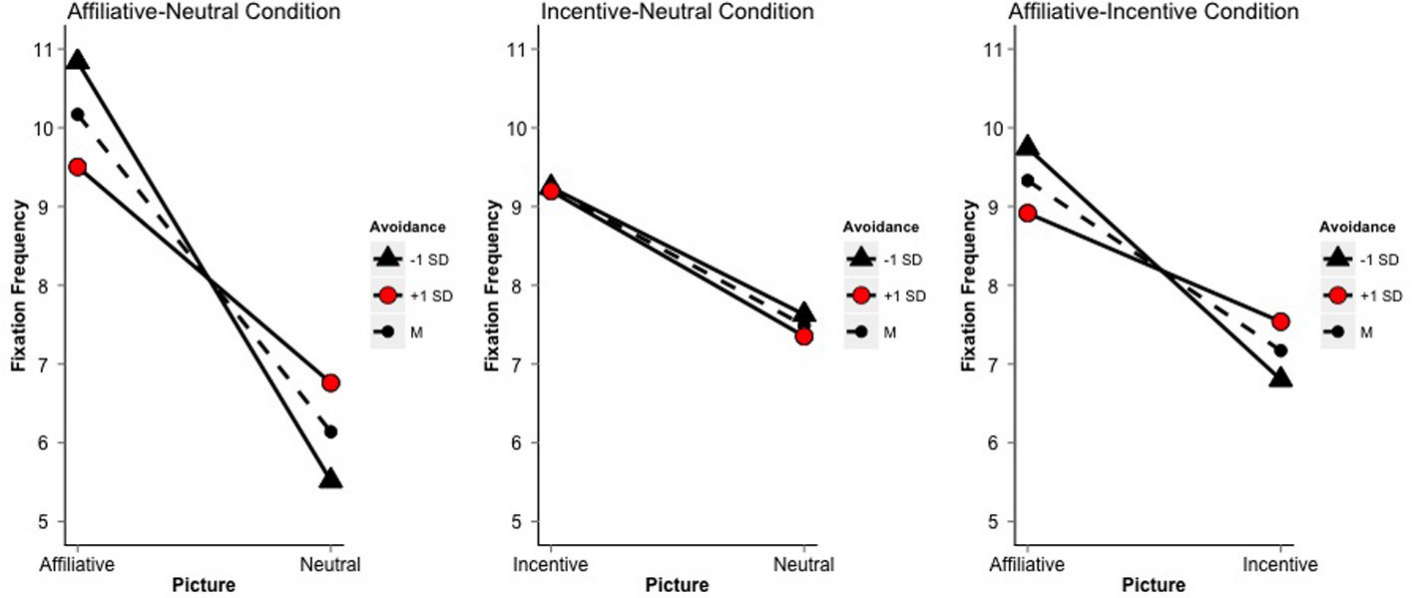
**Simple slopes of picture on fixation frequency for ±1 *SD* and the mean (dashed line) of Avoidance**.

In the affiliative-neutral condition, the repeated measures ANCOVA of fixation frequency × picture-type and attachment indicated that picture-type, anxiety and avoidance, and the interaction of picture-type with each attachment dimension accounted for a significant amount of the variance in number of fixations [*R*^2^_adjusted_ = 0.56, *F*_(5, 108)_ = 30.29, *p* < 0.01]. The interaction of attachment-anxiety × picture-type did not significantly relate to fixation frequencies, but there was a significant interaction of attachment-avoidance and picture-type. Specifically, as shown in Figure 3, simple slopes demonstrated that attachment-avoidance negatively related to fixation frequency on the affiliative picture [*b* = −0.58, *t*_(108)_ = −2.63, *p* < 0.01], but positively related to fixation frequency on the neutral picture [*b* = 0.54, *t*_(108)_ = 2.46, *p* < 0.05]. The simple slopes of picture-type on fixation frequency showed that, on average, for low avoidance scorers (−1 *SD*), there were 5.32 more fixations on the affiliative picture than the neutral picture [*t*_(108)_ = −10.69, *p* < 0.01], while for high avoidance scorers (+1 *SD*), there were only 2.74 more fixations on the affiliative picture than on the neutral picture [*t*_(108)_ = −5.50, *p* < 0.01] (Figure 3). In contrast to SC, as avoidance scores increased, attention was increasingly drawn *away* from the affiliative picture to the neutral picture.

In the incentive-neutral condition, the multiple regression analysis of fixation frequency indicated that picture-type, attachment anxiety and avoidance, and the interaction of picture-type with each attachment dimension accounted for a significant amount of the variance in number of fixations [*R*^2^_adjusted_ = 0.25, *F*_(5, 108)_ = 7.04, *p* < 0.01]. The interaction of avoidance × picture-type was not significant, indicating that the number of fixations on incentive and neutral pictures did not vary by levels of attachment avoidance. However, there was a significant interaction of attachment anxiety with picture type, such that there was a negative relation between anxiety and fixation frequency on incentive pictures [*b* = −0.35, *t*_(108)_ = 3.91, *p* < 0.01], but a non-significant positive relation between anxiety and fixation frequency on neutral pictures [*b* = 0.23, *t*_(108)_ = 1.12, *p* < 0.26]. The simple slopes of picture-type on fixation frequency showed that, on average, for low attachment anxiety scorers (−1 *SD*), there were 2.39 more fixations on the incentive picture relative to the neutral picture [*t*_(108)_ = −5.27, *p* < 0.01], while for high anxiety scorers (+1 *SD*), there were only 1.08 more fixations on the incentive picture than on the neutral picture [*t*_(108)_ = −2.38, *p* < 0.05] (Figure 3). This interaction indicates that as anxiety scores increased, attention was increasingly drawn from incentive to neutral pictures.

In the affiliative-incentive condition, the repeated measures ANCOVA of fixation frequency indicated that picture-type, anxiety and avoidance, and their interaction accounted for a significant amount of the variance in number of fixations [*R*^2^_adjusted_ = 0.29, *F*_(5, 108)_ = 10.30, *p* < 0.01]. There was not a significant interaction of attachment-anxiety and picture-type, but the interaction of attachment-avoidance and picture-type on fixation frequency was significant. As shown in Figure 3, simple slope analyses of attachment-avoidance demonstrated that avoidance negatively but moderately related to fixation frequency on affiliative pictures [*b* = −0.36, *t*_(108)_ = −1.78, *p* = 0.08], but showed no significant relation to fixation frequency on incentive pictures [*b* = 0.31, *t*_(108)_ = 1.53, *p* = 0.13]. This finding suggests that the relation of attachment style to attention is specific to affiliative pictures. The simple slopes of picture-type on fixation frequency demonstrated that for low avoidance scorers (−1 *SD*), there were 2.94 more fixations on affiliative pictures than on incentive pictures [*t*_(108)_ = −6.36, *p* < 0.01], while for high avoidance scorers (+1 *SD*), there were on average only 1.38 more fixations on affiliative pictures than on incentive pictures [*t*_(108)_ = −2.98, *p* < 0.05]. This result indicates that as avoidance scores increased, attention was increasingly drawn away from the affiliative picture to the incentive picture.

### Avoidant attachment as a mediator of SC

In view of the strong association between SC and avoidant attachment, and the association of both of these predictors with attention to affiliative cues, we tested whether the effects of SC on fixations to affiliative cues (relative to neutral cues) were mediated through avoidant attachment (This path was chosen because it was theoretically assumed that SC is a time invariant construct preceding attachment style, which is fluctuates with relationship experiences, and additionally we achieved temporal ordering by assessing SC during recruitment, and avoidance during testing). To build upon the above models, where the effects of SC and avoidance were moderated (in separate models) by picture-type, we performed a moderated mediation model, testing whether there was a *mediated* effect of SC through avoidance on number of fixations that was dependent (*moderation*) on picture-type (affiliative vs. neutral).

**Figure 4:**
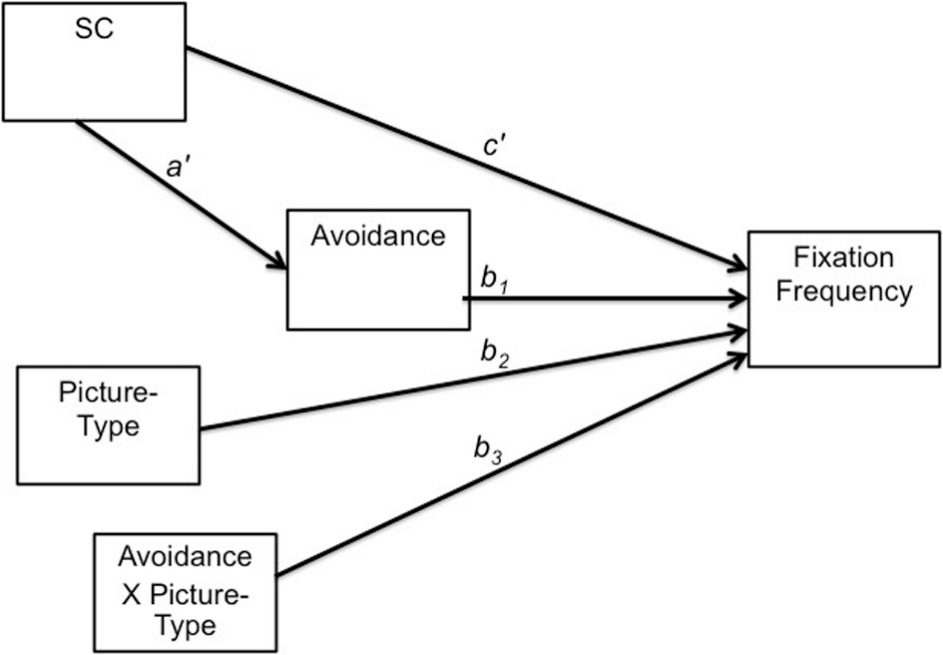
**Moderated mediation model**. See text for details. Estimated directed paths are labeled, where *c*' represents the direct effect of SC on fixation frequency, *a*_1_ the effect of SC on the mediator (avoidance), *b*_1_ the mediated effect of SC on fixation frequency, *b*_2_ the effect of the moderator (picture-type) on fixation frequency, and *b*_3_ the interaction of the mediated effect and picture-type.

Data were analyzed using structural equation modeling in the software program Mplus (Muthén and Muthén, 1998–2010) using Full Information Maximum Likelihood. The model displayed in Figure 4 was parameterized according to Preacher et al. (2007), in which the mediated effect of the predictor on the outcome (*b*_1_) is moderated by another variable, *w*. Thus, *c*' represents the direct effect of SC on fixation frequency, *a*_1_ the effect of SC on the mediator (avoidance), *b*_1_ the mediated effect of SC on fixation frequency, *b*_2_ the effect of the moderator (picture-type) on fixation frequency, and *b*_3_ the interaction of the mediated effect and picture-type.

Of interest to the test for moderated mediation is the *conditional indirect effect* of SC through avoidance on affiliative fixations. In mediation analysis, the indirect effect is the magnitude of the effect of the predictor through the mediator on the outcome (*a*_1_ × *b*_1_), indicating the presence (or absence) of a mediated effect. In *moderated* mediation, the indirect effect is conditional upon levels of another variable, in this case, the indirect effect of SC through avoidance on fixation frequency being conditional upon picture-type, as SC and avoidance are hypothesized to modulate attention to affiliative cues, specifically. Therefore, we expected a significant indirect effect for the affiliative picture, but not for the neutral picture. Conditional indirect effects are calculated by multiplying the effect of the predictor on the mediator by the sum of the mediated effect, and the interaction effect at the level of picture-type; *a*_1_ × (*b*_1_ + *b*_3_ × Picture-Type).

Results are displayed in Table 4. SC and avoidance were standardized for ease of interpretation, and bias corrected bootstrap confidence intervals were calculated for all estimates [*k* = 5000 repetitions, see MacKinnon et al. (2004) and Preacher et al. (2007) on the use of bootstrapping in mediation analyses to improve accuracy]. To assess the presence of simple mediation effects, avoidance was dropped from the model to gauge the direct effect of SC in the absence of the mediator. The necessary conditions to establish mediation were met: (a) SC and avoidance were significantly related (see *a*'); (b) SC had a significant direct effect on fixation frequency (*b* = 0.56, *p* < 0.05, calculated by dropping avoidance from the model); (c) avoidance and fixation frequency were significantly related (see *b*_1_); and (d) when mediated by avoidance, the direct effect of SC was no longer statistically significant (*b* = −0.34, *p* > 0.05, see *c*'), suggesting full mediation of the direct effect of SC on fixation frequency by avoidance (Baron and Kenny, 1986).

**Table 4:** **Moderated mediation results**.

**Parameter**	**Regression coefficient**	***SE***	**95% Confidence interval**
*a*'	−0.68^**^	0.07	(−0.82, −0.55)
*c*'	−0.34	0.23	(−0.79, 0.12)
*b*_1_	−0.94^**^	0.33	(−1.63, −0.36)
*b*_2_	−4.03^**^	0.34	(−4.72, −3.36)
*b*_3_	1.29^**^	0.32	(0.68, 1.95)
Conditional indirect effect (picture = affiliative)	0.64^**^	0.23	(0.24, 1.15)
Conditional indirect effect (picture = neutral)	−0.24	0.17	(−0.58, 0.07)
χ ^2^, *df, p*	221.83, 7, 0.00		

** *p < 0.01. Parameters identified as presented in Figure 4. Bootstrap confidence interval calculated by sampling with replacement, k = 5000 repetitions*.

Moreover, the moderated mediation model supported that the indirect effect of SC on fixation frequency depended on picture-type. That is, the conditional indirect effect of SC through avoidance for *affiliative* pictures was significant (*b* = 0.64, *p* < 0.01), but the conditional indirect effect for *neutral* pictures was not significant (*b* = −0.24, *p* > 0.05). These results support the notion that the positive association between SC and fixations on affiliative cues is mediated by levels of attachment avoidance. To calculate a gauge of the effect size of the moderated mediation effect, we used the mediation function from the MBESS (Kelley and Lai, 2010) R package. As recommended by Preacher and Kelley (2011), we report *K*^2^, a ratio of the amount of variance in the outcome accounted for by the indirect effect to the maximum variance in the outcome that could possibly be accounted for by the indirect effect (as the possible accountable variance is bounded by the covariance matrix). Thus, *K*^2^ = 0 indicates an absence of a linear indirect effect, and *K*^2^ = 1 indicates that the indirect effect is as large as it could be. For the conditional indirect effect of SC through avoidance on affiliative pictures, *K*^2^ = 0.15. In contrast, for the neutral picture, *K*^2^ = 0.00. This result again indicates a modest indirect effect of SC through avoidant attachment on attention, which depends on the affiliative nature of the stimulus.

## Discussion

At the broadest level, the current study demonstrates that variation in trait SC and avoidant attachment significantly modulated the degree of attention directed specifically to affiliative cues, suggesting that these contributors to social valuation influence the salience assigned to social contexts connoting closeness and intimacy. In particular, trait levels of SC positively related to attention on affiliative pictures, when those pictures were viewed alongside neutral pictures, as well as strong incentive stimuli. For higher SC individuals, there were significantly more fixations on affiliative pictures, and significantly less fixations on neutral or incentive pictures than for lower SC individuals. Importantly, SC did not relate to attention to incentive stimuli when those cues were paired with neutral pictures, indicating that the SC influence on attention is specific to affiliative cues.

These latter findings are consistent with the notion that personality traits modulate salience encoding specifically of the environmental cues that elicit a trait's respective underlying neurobehavioral emotional system (Depue and Collins, 1999; Gray and McNaughton, 2000; Depue and Morrone-Strupinsky, 2005). In the case of SC, this suggests that individuals who value social relationships and experience pleasure from interpersonal contact will have established historically an enhanced salience encoding of affiliative cues, and that this enhanced encoding influences attentional mechanisms toward affiliative cues on a contemporaneous basis.

Our results also suggest that attachment avoidance is an important contributor to attentional engagement with affiliative stimuli, despite these cues being unrelated to any particular attachment figures. This conclusion is evidenced by a negative relationship between avoidance and fixation frequency on affiliative pictures when presented alongside neutral or incentive stimuli, and lower average fixation frequency on affiliative cues among highly avoidant individuals. This finding is consistent with prior reports of associations between avoidance and cognitive suppression of *social* information, including attachment-related words, information about romantic partners, and angry and happy faces (Dykas and Cassidy, 2011). Importantly, attachment anxiety did not relate significantly to attention to affiliative cues, so the relation of attachment style to attention to affiliative cues (unrelated to a particular attachment figure) appears to be specific to avoidant attachment (see further discussion below).

A novel finding in our results demonstrates that the variance in SC that predicts attention to affiliative cues is significantly mediated by attachment avoidance. This raises the question of what is the nature of the distinctive contribution to attention by SC and avoidant attachment? With respect to SC, we and others have hypothesized that the neurobehavioral emotional system that underlies trait SC relates to the capacity to experience consummatory reward elicited by affiliative cues, especially soft tactile stimulation (Depue and Morrone-Strupinsky, 2005; Machin and Dunbar, 2011). Consummatory reward is mediated by the μ-opiate system when the latter is activated by the consumption of natural rewarding stimuli, such as palatable food and sexual objects, and social stimuli (including soft touch), which gain their rewarding value also through the neural operations in this same system (Depue and Morrone-Strupinsky, 2005; Machin and Dunbar, 2011). Individual differences in SC may, accordingly, be conceived as reflecting, at least in part, variation in the capacity to experience consummatory reward when elicited by social stimuli. Recently, the notion that variation in sensitivity to social cues in humans emerges early in development was supported by a study assessing physiological and behavioral reactivity to pleasant, soft touch in 9-month-old infants (Fairhurst et al., 2014).

Across development, heightened consummatory reward in response to interpersonal exchanges yields an encoded affiliative value for social stimuli, and builds upon the array of environmental cues capable of activating the affiliative system. Through such processes, unconditioned and conditioned social stimuli become organized as encoded memory networks that represent the general context and specific features associated with consummatory reward (Depue and Fu, 2013). Theoretically, SC would modify the magnitude of the encoded value of memory networks, which would be reflected in differential encoding of affiliative cues in the BLA. Such differential encoding would subsequently drive attentional processes via extensive BLA backprojections to cortical and subcortical brain regions that regulate perceptual and attentional processes (Tamietto and de Gelder, 2010), thus fostering variation in affiliative behavioral tendencies.

In contrast to SC, attachment avoidance is thought to arise as an adaptive strategy for dealing with insensitive parenting behavior from the primary caregiver referred to as “rejecting” due to displays of disinterest, hostility and criticism, and little affective expression or physical contact (Ainsworth et al., 1978). Longitudinally, avoidantly-attached infants continue to display emotional distance, and distrust and withdraw from others across childhood (George and Main, 1979; Sroufe, 1983; Sroufe et al., 1983) and into adulthood (Sroufe, 2005). Thus, an avoidant attachment style nurtured by rejecting primary caregivers is expected to thwart the developmental experiences required to learn the rewarding value of closeness and intimacy.

More broadly, both SC and attachment patterns contribute to individual differences in the valuation of closeness and intimacy. Consistently, agreeableness and warmth (a facet of extraversion) assessed by the NEO Personality Inventory, which are both associated with SC (Tellegen and Waller, 2008), are negatively correlated with attachment avoidance (Shaver and Brennan, 1992). According to behavioral genetics research, the variance common to attachment patterns and personality is attributable to genetic factors, whereas environmental factors contributing to attachment style are largely independent of personality (Donnellan et al., 2008). These results raise the possibility that neurobehavioral mechanisms putatively underlying SC, which contribute in part to the valuation of interpersonal cues, account for the association between personality and attachment, and that attachment dimensions are uniquely refined by particular attachment-related experiences with caregivers and close others.

Experiences with attachment figures across development are theorized to shape how one processes attachment-related social information, particularly when presented with attachment-related information that previously led to suffering (Dykas and Cassidy, 2011). For avoidant individuals, positive as well as negative social cues can activate painful feelings associated with rejection, neglect, and the absence of closeness and comfort, leading to a deactivation strategy from social information to filter such cues from conscious awareness. The theorized role of subconscious, subcortical mechanisms in biasing the processing of affiliative cues that are reflected in trait SC might be mediated through such attachment-related top-down cognitive-control self-schemas.

Thus, the finding that attachment avoidance averts attention from affiliative cues suggests that the value assigned to affiliative cues is a product of experiences with attachment figures as well as neurobiological processes underlying variation in trait levels of SC. Perhaps SC provides a more discrete assessment of the value assigned to closeness that shapes responsiveness to both affiliative cues as well as the broadening contexts that elicit the affiliative system across development in the absence of severe attachment difficulties. Avoidance could jointly reflect the responsiveness of the affiliative system to relevant cues as modified by previous relationship experiences. More specifically, attentional biasing to affiliative cues due to the consummatory reward experienced from close interpersonal contact could be *compromised* by a series of rejecting relationships, which could alter the valuation of closeness and intimacy. Thus, attachment avoidance may be viewed as a higher-order construct that is influenced by both neurobehavioral encoding of affiliative cues and the personal social experiences that modify the expression of the neurobehavioral system in close social relationships. In this case, we suggest that neurobehavioral processes on social encoding captured by SC influence attention *through* their manifestation into avoidant attachment, as ongoing attachment relations modify how interpersonal cues are encoded and responded to.

There are two notable limitations to this study. First, it is not possible to parse the effects of relationship histories from natural dispositions for experiencing social reward with our self-report measurements of SC and attachment style, so we must emphasize that much of our interpretation of the mediation findings is driven by theoretical assumptions. Our theoretical model could be further substantiated by an experimental or longitudinal study assessing whether and in which ways particular attachment experiences influence the value attributed to affiliative cues and biased attentional processing. For example, a parenting intervention allowing for the manipulation of attachment experiences could capture causal effects of these experiences on the affective encoding of social cues, or a longitudinal study could follow trajectories of individuals with varied sensitivities to close social contact in relation to qualities of ongoing attachment relationships. Finally, our sample of female college students limits the generalizability of these findings, warranting replication in more representative samples.

In conclusion, our findings demonstrate a strong link between trait levels of SC and avoidant attachment in influencing attention specifically to affiliative cues. These results provide novel evidence for the involvement of biased attentional processing of affiliative cues in the behavioral tendency to value and engage in close, interpersonal ties. Thus, theoretically, the broader construct of affiliation (including SC and attachment avoidance) may be viewed as reflecting a distinct, multivariate emotional system that evolved to respond to and bias attention toward salient social cues necessary for the formation and maintenance of social bonds key to survival.

### Conflict of interest statement

The authors declare that the research was conducted in the absence of any commercial or financial relationships that could be construed as a potential conflict of interest.
